# Effect of *Lb. plantarum* BFE 5092 Fermentation on Antinutrient and Oligosaccharide Composition of Whole Red Haricot Bean (*Phaseolus vulgaris L*)

**DOI:** 10.1155/2020/8876394

**Published:** 2020-08-10

**Authors:** Vivian C. Kitum, Peter K. Kinyanjui, Julius M. Mathara, Daniel N. Sila

**Affiliations:** Department of Food Science, Jomo Kenyatta University of Agriculture and technology, Nairobi, P.O. Box 62000-00200, Nairobi, Kenya

## Abstract

Common beans are a leguminous plant of the genus Phaseolus. They are rich in protein, energy, and minerals. They confer a wide range of health benefits when consumed. Utilization of common bean has however been poor due to high antinutrient content that results in reduced nutrient digestibility and mineral bioavailability. Flatulence after consumption is also a huge deterrent to common bean consumption. Lactic acid fermentation is the most common form of food fermentation with the Lactobacilli spp dominating most spontaneous fermentations. The objective of this study was to determine the effect of lactic acid bacteria (LAB) on the antinutrient and flatulence causing oligosaccharide composition of red haricot bean. A factorial research design was used in the study. Red haricot beans were sorted and soaked for 15 h. The soaked beans were fermented in 2% salt-sugar solutions for 120 h. Experimental batch was inoculated with Lb. plantarum BFE 5092 (IF), and the control batch was spontaneously fermented (SF). Microbial growth and pH were monitored every 24 h during fermentation. After fermentation, the beans were dried and milled, and the flours were subjected to biochemical analysis. ANOVA was done using SPSS statistics 23. The pH decreased significantly (*P* < 0.05) from 6.06 to 3.9 in both batches at the end of fermentation. The LAB counts significantly increased (*P* < 0.05) in both batches, whereas coliform counts decreased significantly (*P* < 0.05). Fungi were not detected in both batches. Soaking lowered tannins and phytates and raffinose concentrations significantly but had no significant effect on stachyose concentration. At the end of 120 h of fermentation, the tannin content was 109.50 and 54.04 mg/100 g in IF and SF, respectively. Phytates were at 242.52 and 163.43 mg/100 g in IF and SF, respectively. Raffinose content was 32.85 and 32.58 mg/100 g in IF and SF, respectively, while stachyose content was 593.33 and 467.49 mg/100 g in IF and SF, respectively. This research showed that LAB is able to ferment soaked whole red haricot and lower the tannin, phytate, raffinose, and stachyose content significantly. Spontaneous fermentation lowered these antinutrients and oligosaccharides better than inoculation with Lb. plantarum BFE 5092.

## 1. Introduction

The year 2016 was declared an international year of pulses by the United Nations (UN) to increase public awareness on the benefits of consuming pulses. Pulses are edible seeds of the legume family in which common beans belong. They are widely grown around the world, hence available and affordable. Common beans are a good source of protein, rich in lysine [1] the limiting amino acid in cereal grains. They are also rich in essential minerals [2] providing a solution to hidden hunger which plagues many. They have also been associated with numerous health benefits [3] such as lowering blood cholesterol, stabilizing blood sugars, and alleviating constipation.

However, though beans are nutritionally rich, they are also high in antinutrients [4]. These are biologically active compounds that, when consumed reduce protein and starch digestibility, make minerals nonbioavailable [5]. The antinutrients in common beans include phytates which lowers mineral bioavailability and tannins which affect nutrient digestibility. These antinutrients lower the potential of common beans to meet the nutritional needs of the populations that heavily depend on them as an affordable source of protein and minerals [6]. In addition, common beans have high raffinose family oligosaccharides (RFOs) content which causes flatulence [6] after consumption. Flatulence results in stomach discomforts and passing of wind which are social problems associated with common bean consumption. These lead to the shunning of common bean by consumers denying them the nutrition benefits of common beans.

In a bid to lower the antinutrients and oligosaccharides in beans, researchers like Adewumi and Odunfa [7], Granito et al. [8], Tope [9], and Worku and Sahu [10] have fermented different beans. Fermentation is a metabolic process in which energy and carbon is derived from an organic compound [11] carried out by fermentation microorganisms. Fermented foods account for about 25% of the total foods consumed around the world [12]. Most of these foods are prepared at household levels using natural fermentation methods. Lactic acid bacteria (LAB) have been isolated as the major microorganisms in many of the naturally fermented food products [13]. Studies by Adewumi and Odunfa [7], Granito and Alvarez [14], Granito et al. [8], Worku and Sahu [10], and other workers show that fermentation lowers antinutritional compounds in common beans. However, a majority of these studies have been applied to bean flour slurry, yet in East Africa, common beans are mostly consumed as whole grain. Therefore, populations continue to grapple with reduced nutrient digestibility and bioavailability as well as flatulence after consuming common bean. A good proportion of the population avoid its consumption all together. This study therefore seeks to scale up research and establish ways to ferment whole common beans in order to increase its consumption and unlock its nutritional potential.

## 2. Materials and Methods

### 2.1. Materials

Raw red haricot beans (Wairimu) were acquired from the National Cereals and Produce Board (NCPB) Nairobi, Kenya. *Lb. plantarum* BFE 5092 strain used was isolated by Maina et al., [15]. The table salt (Kensalt) and local sugar (Kabras) used in this study were purchased locally. The media used for microbial determination was analytical grade from Himedia, Mumbai, India.

### 2.2. Methods

#### 2.2.1. Sample Preparation

The red haricot beans were hand sorted to remove dirt and defective grains. About 500 g of the beans was drawn as control sample, milled raw and stored in freezer awaiting biochemical analysis.

#### 2.2.2. Soaking of the Red Haricot Beans

About 1.5 kg of the sorted red haricot beans was washed in distilled water, and all floats were removed. The beans were then soaked in distilled water at a ratio of 1 : 5 weight per volume (*w*/*v*) for 15 h at room temperature. The soaking water was discarded, and beans were rinsed in distilled water. About 200 g portion of the soaked red haricot beans was dried at 60°C for 10 h in an oven, milled then stored in a freezer awaiting biochemical analysis (soaked sample).

#### 2.2.3. Preparation of Fermentation Solutions

Fermentation solution (2% salt-sugar solution) was prepared by dissolving 12 g of salt and sugar 1 : 1 *w*/*w* in six fermentation bottles containing 600 ml of distilled water each. The bottles with the fermentation solutions were sterilized by autoclaving at 121°C for 15 min then allowed to cool to room temperature before use.

#### 2.2.4. Starter Culture Preparation

A pure colony of *Lb. plantarum* BFE 5092 was transferred to DeMann Ragosa Sharpe (MRS) broth and incubated for 24 h at 30°C. Approximately, 0.6 ml of broth was then transferred into 1.5 ml Eppendorf tubes and vortexed at 13,000 rpm for 5 min. The supernatant was discarded and the pellet dissolved in 600 *μ*l sterile ringer solution.

#### 2.2.5. Fermentation of Soaked Beans

About 200 g of soaked beans, from above, was transferred to each fermentation bottle containing 600 ml of 2% salt-sugar fermentation solution prepared above. Exactly, 600 *μ*l of *Lb. plantarum* BFE 5092 starter culture prepared above was then added to three of these fermentation bottles. The bottles were labelled 24 h inoculated fermentation (IF), 72 hr IF and 120 h IF. The remaining 3 bottles were labelled 24 h spontaneous fermentation (SF), 72 h SF and 120 h SF.

The fermentation bottles were then left on a sterile bench to ferment at 25 ± 2°C for 120 h. Brine samples were drawn aseptically using a pipette and sterile pipette tips at 0 h, 24 h, 72 h, and 120 h for pH determination and microbial enumeration.

#### 2.2.6. pH Determination

Approximately, 5 ml of the fermentation solution was drawn aseptically after every 24 h of fermentation for pH determination using a pH meter (HI 2211, Hanna Instruments, Japan).

#### 2.2.7. Microbial Enumeration

Conventional microbiological methods were used for microbial enumeration. Spread plate method was used. Each analysis was carried out in triplicate. All bacterial and fungal counts were expressed as colony-forming units per millilitre (CFU/ml).

#### 2.2.8. Sample Preparation for Biochemical Tests

At the end of each fermentation, the fermentation solution was discarded, and the fermented red haricot bean (*Phaseolus vulgaris L*) was spread on clean trays. They were then dried in an oven at 60°C for 10 h. The dried beans were then milled, and the resulting sample flour was stored at 4°C in polyethylene bags awaiting biochemical analysis.

#### 2.2.9. Determination of Tannin Content of Red Haricot (*Phaseolus vulgaris L*.) Bean Flours on Dry Weight Basis

Tannin content was determined using the Vanilin-HCL method of Price et al. (1978) using about 0.2 g of bean flour sample. The absorbance of the sample extracts and standard solutions were read at 500 nanometers using a UV-vis photospectrophotometer (UV mini 1240 model, Shimadzu, Japan).

#### 2.2.10. Determination of Phytate Content of Red Haricot (*Phaseolus vulgaris L*.) Bean Flours on Dry Weight Basis

Phytates were extracted using the method of Camire and Clydesdale. (2006) with modification. About 0.5 g of milled bean flours were extracted. Liquid chromatography-mass spectrophotometry (LC-MS) (Genevac, DNA-23050-A00, England) analysis was done using Shimadzu Refractive Index Detector (RID 6A). The mobile phase was 0.005 N sodium acetate in distilled water at a flowrate of 0.5 *μ*l/minute.

#### 2.2.11. Raffinose Family Oligosaccharide Extraction and Quantification

Quantification of raffinose and stachyose was done using the method of Antonio et al. [16].

### 2.3. Statistical Analysis

Each analysis was done in triplicate and the experiments conducted three times. Data was presented as means ± standard error of means (SEM) or standard deviation (SD) of three separate determinations. Contrast ANOVA was conducted as well as pairwise comparison of estimated marginal means at *P* ≤ 0.05 using LSD. Statistical analysis was carried out using SPSS statistics version 23.

## 3. Results and Discussion

### 3.1. Effect of Soaked Whole Red Haricot Bean (*Phaseolus vulgaris L*) Fermentation on pH

**Figure 1:**
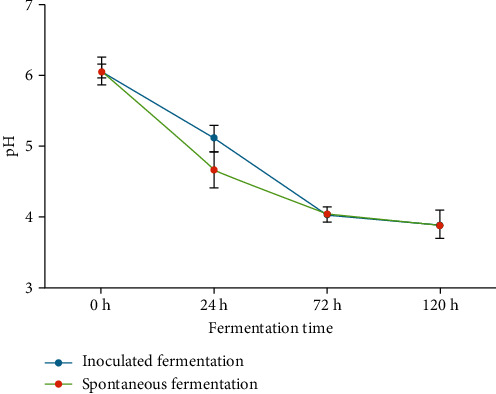
Effect of fermentation time on the pH during the fermentation of soaked whole red haricot bean presented as pH ± SEM (*n* = 3).

The changes in pH of the fermentation solutions during the fermentation of soaked whole red haricot bean are presented in Figure 1. The pH of the fermentation solution at the beginning of fermentation was 6.06. Fermentation of the beans resulted in a significant decrease (*P* < 0.0001) in pH in both the inoculated and spontaneously fermented batches. After 24 h of fermentation, a pH of 5.1 and 4.7 was recorded in the IF and SF batches, respectively. This phenomenon is similar to the findings of Granito et al. [8] who reported a pH of 4.45 and 4.10 for the inoculated and spontaneous batches, respectively. The differences in values could be attributed to different legume varieties used in the studies. In this study, red haricot bean was used, whereas Granito et al. [8] used *P. vulgaris* Victoria variety.

The fermenting microorganisms were able to break down fermentable sugars in the beans [17] producing organic acid as by-products. This resulted in the decrease in pH during the fermentation of the red haricot beans. The pH continued to decrease significantly with an increase in fermentation time in both batches. At the end of 120 h of fermentation, a pH of 3.9 was recorded in both the IF and SF batches. This reduction in pH is comparable to the change from 6.24 to 3.87 reported by Onwurafor et al. [18] after 72 h fermentation of mung bean. The difference in time could be attributed to specie and varietal difference of the legumes used.

### 3.2. Microbial Growth during the Fermentation of Soaked Whole Red Haricot Bean (*Phaseolus vulgaris* L.)

The growth of total viable count during fermentation of soaked whole red haricot bean is presented in Table 1. The total viable count increased significantly (*P* < 0.0001) within the first 24 h of fermentation in the IF batch from log_10_ 6.8 CFU/ml to log_10_ 8.6 CFU/ml. Total viable count then declined significantly (*P* < 0.001) with increased fermentation time in the IF batch to log_10_ 8.3 CFU/ml after 120 h of fermentation. In the SF batch, there was a significant increase (*P* < 0.0001) in the total viable counts after 24 h of fermentation from log_10_ 4.8 CFU/ml and log_10_ 8.0 CFU/ml. Increased fermentation time resulted in further significant increase of total viable count to log_10_ 9.4 at the end of 120 h of fermentation. These counts were higher in comparison to the IF batch and were comparable with the LAB counts in the uninoculated batch.

LAB dominated the fermentation of soaked whole red haricot bean after 24 h of fermentation (Table 1); this is in agreement with [14]. The growth of LAB increased significantly (*P* < 0.0001) with an increase in fermentation time in the inoculated batch log_10_ 6.5 CFU/ml to and log_10_ 8.6 CFU/ml, respectively, after 24 h of fermentation.

The LAB counts then decreased to log_10_ 8.3 CFU/ml after 72 h of fermentation and remained unchanged by the end of 120 h of fermentation. In the SF batch, there was a significant increase (*P* < 0.0001) in the LAB counts after 24 h of fermentation from log_10_ 4.0 CFU/ml to log_10_ 7.85 CFU/ml. Increased fermentation time resulted in increased growth of LAB to log_10_ 9.4 CFU/ml at the end of 120 h of fermentation. The higher counts of LAB in both the IF and SF batches is an indicator that both the *Lb. Plantarum* BFE 5092 and the preexisting LAB are able to utilize whole soaked bean as a substrate and proliferate [13]. The table sugar in the fermentation solution could also have provided an extra energy source for the bacteria's metabolism hence accelerated growth [19].

At the start of fermentation, the coliform count was at log_10_ 4 CFU/ml (Table 1). This is comparable to the findings of Granito and Alvarez [14] who reported 62% of total aerobic count to be coliforms at the start of black bean fermentation. In the inoculated batch, a significant increase (*P* < 0.0001) in the coliform count was recorded after 24 h of fermentation from log_10_ 4 to log_10_ 8.1. This was followed by a significant reduction (*P* < 0.0001) in the number of coliforms to log_10_ 5.5 CFU/ml and log_10_ 3.2 CFU/ml after 72 h and 120 h of fermentation. A similar trend was observed in the spontaneously fermented batch. A significant increase (*P* < 0.0001) in coliform count from log_10_ 4.6 CFU/ml to log_10_ 8.1 CFU/ml was made after 24 h of fermentation. This was then followed by a significant decrease in the coliform count to log_10_ 6.5 CFU/ml and log_10_ 3.6 CFU/ml after 72 h and 120 h of fermentation. The significant decrease in the coliform counts in the batches during fermentation is attributable to the acid production by lactic acid bacteria [14]; this was evidenced by low pH (Figure 1) in these batches. Coliforms are sensitive to low pH and are inhibited from pH below 4.4 [18]. The pH of 3.9 in the batches (Figure 1) is lower than the pH 4.4 from where the coliform growth is inhibited. This explains the decreased coliform count after 72 h of fermentation.

Fungi was not detected in all the inoculated and spontaneous fermentation batches. This observation is similar to the findings of Granito and Alvarez [14] after the fermentation of black beans for 48 h.

### 3.3. Effect of Fermentation on Antinutrient Content of Soaked Whole Red Haricot Bean (*Phaseolus vulgaris* L)

#### 3.3.1. Tannins

The tannin content in the raw red haricot bean (*Phaseolus vulgaris* L) was 333.68 mg/100 g (Table 2). This was lower compared to the 4533 mg/100 g reported by Chaudhary and Sharma [3] and higher than 210 mg/100 g reported by Olanipekun et al. [20] tannin content in whole red kidney beans. The difference could be associated with varietal differences of the beans. When the whole red haricot beans were soaked, tannin content reduced significantly (*P* < 0.0001) to 306.82 mg/100 g. The reduction is in agreement with the findings of Reddy et al. [21], Ferreira et al. [22], and Fernandes et al. [23] who observed that soaking of beans in water and discarding the water eliminates a percentage of tannins. Kamel et al. [24] reported a 74.73% decrease in tannin content of black bean after 12 h. In this present study, the decrease was 8.4%, and the difference could be attributed to the difference in bean varieties used and that Kamel et al. [24] changed the soaking water twice. This is as a result of leaching out of the tannins into the soaking water [3, 25].

Fermentation resulted in a decrease in tannin content in all the fermentation batches. In the IF batch, the tannin content reduced from 306.82 mg/100 g in the soaked whole bean to 283.41 mg/100 g after 24 h of fermentation. Higher tannin reduction in the IF batches occurred between 72 h and 120 h of fermentation. At the end of fermentation, the tannin content in the IF batch was 109.50 mg/100 g. In the SF batch, higher tannin losses occurred compared to the batches inoculated with *Lb. plantarum* BFE 5092. The tannin content decreased from 306.82 mg/100 g to 201.59 mg/100 g after 24 h of fermentation. Tannin losses were the highest between 24 h and 72 h of fermentation. At the end of spontaneous fermentation, the tannin content was 54.04 mg/100 g. Fermentation significantly reduced the tannin content in both the IF and SF batches. This is in agreement with Granito and Alvarez [14] and Adeniran et al. [5] who reported an 83%, 89.5%, and 68.42% decrease in tannin content of cooked fermented black beans, lima bean, and locust bean, respectively. The reduction of tannin as a result of fermentation can be attributed to the hydrolysis of polyphenolic compounds of tannin complexes during fermentation [5]. In this current study, it was observed that the SF batches lowered tannin content more compared to the batches inoculated with *Lb. plantarum* BFE 5092. This could indicate that the bacteria present in the spontaneous fermentation batches were more adapted to the hydrolysis of tannin complex in comparison to *Lb. plantarum* BFE 5092. Between 24 h and 120 h of fermentation, *Lb. plantarum* BFE 5092, however, is able to break down appreciable amounts of tannins.

#### 3.3.2. Phytates

The phytate content in raw red haricot bean was 482.99 mg/100 g as presented in Table 2. This was lower compared to the 543 mg/100 g reported by Chaudhary and Sharma [3] for red kidney beans and could be due to varietal difference of the beans. When the whole red haricot beans were soaked, the phytate content reduced significantly (*P* < 0.0001) to 387.25 mg/100 g. The reduction of phytates in the beans after soaking is in agreement with Reddy et al. [21], Ferreira et al. [22], and Fernandes et al. [23] who established that soaking of beans in water and discarding the water eliminates a percentage of phytates. This was also observed in soybean [26] and in red kidney beans [3] due to leaching of phytate ions into the soaking, because the phytate ion is water-soluble in nature [3, 25, 27]. Nakitto et al. [27] also reported that the imbibition of water also activates phytase enzymes in the beans which breaks down of phytates. A combination of these effects contributes to the decrease in phytate content during soaking. A further decrease in phytate concentration was observed when the beans were fermented (Table 2). In the IF batch, phytate concentration significantly decreased (*P* < 0.0001) from 387.25 mg/100 g to 242.52 mg/100 g at the end of fermentation. The rate of phytate loss in this batch increased with fermentation time. The highest losses were observed between 72 and 120 h of fermentation. In the SF batch, a significant decrease (*P* < 0.0001) in phytate concentration was observed at the onset of fermentation. The phytate content in this batch decreased from 387.25 mg/100 g to 279.34 mg/100 g after 24 h of fermentation. The rate of phytate loss in this batch then decreased with an increase in fermentation time. The decrease in phytate concentration as a result of fermentation of whole red haricot beans established by this current study is similar to the findings of Adeniran et al. [5] who reported a 77.82% and 73.53% reduction of phytate in lima and locust bean, respectively. The highest phytate loss in the SF batch was between 0 h and 24 h of SF of the beans when the pH was between 6.06 and 4.65 (Figure 1). This pH could have favoured the activity of cereal phytase [28] and extracellular phytase enzymes from preexisting LAB [29]. A decrease in pH may have denatured the phytase enzymes [28] thus reducing the rate of phytate hydrolysis in the SF batch as fermentation time increased. In the IF batch, the highest phytate losses occurred between 72 h and 120 h of fermentation when the pH was between 4.01 and 3.88 (Figure 1). This is in agreement with Mohamed et al. [25] who reported an increased rate of phytate loss with increased fermentation time of kidney beans. Mohamed et al. [25] postulated that lowered pH favoured phytase activity. The difference in the rate of phytate loss in the IF and SF in this study could be attributed to the adaptation of the bacteria in the spontaneous fermentation to cereal fermentation compared to the *Lb. plantarum* BFE 5092 which was isolated from fermented milk by Maina et al. [15]. The reduction of phytate during fermentation of soaked whole red haricot bean can be attributed to the activity of phytase enzyme from the beans and fermenting microorganisms [5, 30–32] and from passive diffusion of water-soluble phytate into the fermentation solution [28]. This could be the reason why losses in phytate was observed in the IF and SF batches even after pH was lowered below 5.1. Loss of phytic improves the availability of minerals as they become available for utilization by the digestive system of the consumer.

### 3.4. Effect of Fermentation on Oligosaccharide Content of Soaked Whole Red Haricot Bean (*Phaseolus vulgaris* L)

#### 3.4.1. Raffinose

The concentration of raffinose in the raw red haricot bean (*Phaseolus vulgaris* L) was 72.27 mg/100 g (Table 2). This was between the range of 69 and 429 mg/100 g reported by Agbenorhevi et al. [33] for cowpea. Soaking of the red haricot bean resulted in a significant decrease (*P* < 0.05) in raffinose concentration from 72.27 mg/100 g to 55.91 mg/100 g (22.64% decrease). This agrees with the findings of Agbenorhevi et al. [33] who reported 52-210 mg/100 g raffinose concentration in soaked cowpea, which accounted for 18.2%-53.6% loss in raffinose concentration. Agbenorhevi et al. [33] soaked the cowpea in distilled water at 25°C conditions similar to those in this present study. Nyombaire et al. [6] reported an up to 80% decrease in raffinose content of soaked red kidney beans after 12 h of soaking. This high decrease and difference with the present study could be attributed to the different soaking conditions used by the researchers. Nyombaire et al. [6] soaked the beans in distilled water with sodium bicarbonate and sodium polyphosphate at 77° C. Agbenorhevi et al. [33] suggested that extent of oligosaccharide loss is varied due to varietal differences which affect the degree of absorption of water, which also explains the difference in the % decrease in raffinose content in the current study in comparison to the findings of other researchers. The reduction of oligosaccharides during soaking is attributable to their solubility in water. When the soak water is absorbed by the seeds during soaking, the oligosaccharides dissolve and are leached into the soak water [6, 33, 34].

Fermentation resulted in a decrease in raffinose concentration in both the fermentation batches (Table 3). The raffinose concentration decreased from 55.91 mg/100 g to 50.58 mg/100 g (9.52% decrease) after 24 h of fermentation in the IF batch. Increased fermentation time resulted in a further decrease in the raffinose concentration of the beans in this batch. The highest decrease of 26.85% occurred between 72 h and 120 h of fermentation lowering the raffinose content to 32.85 mg/100 g. A similar trend was observed in the SF batch, raffinose content decreased from 55.91 mg/100 g in the soaked bean to 50.22 mg/100 g (10.16% decrease) after 24 h of fermentation. Increased fermentation time resulted in further decrease in the raffinose concentration. At the end of 120 h of fermentation, the raffinose concentration had decreased to 32.58 mg/100 g. In the fermentation batches, it was observed that the rate of raffinose reduction increased with fermentation time. The highest decrease occurring between 72 h and 120 h of fermentation. This was contrary to the findings of Adewumi and Odunfa [7] who reported that raffinose content decreased significantly between 24 h and 72 h of fermentation of *Vigna unguiculata* beans with *Lb. plantarum.* Kumar et al. [35] also reported that *α*-galactosidase activity was detected after 12 h incubation with maximum activity at 72 h beyond which production and activity of the enzyme declined. Carević et al. [36] and Carrera-silva et al. [37] also reported that *α*-galactosidase enzyme had an optimum pH of 4.3-5.0. The findings in this study were different since the rate of raffinose reduction increased with fermentation time (Table 3). The higher rate of raffinose loss after 120 h could be attributed to the difference in the fermenting microbes [38]. It could also be attributed to the growth of LAB in these batches which were still increasing in number even after 72 h of fermentation (Table 1).

#### 3.4.2. Stachyose

The stachyose content of raw red haricot bean was 1264.45 mg/100 g (Table 3). This was lower compared to 2400 mg/100 g reported by Rupe [39] for red bean and higher compared to 109-570 mg/100 g reported by Agbenorhevi et al. [33] for cowpea. The high concentration of stachyose in red haricot bean agrees with the report of Granito et al. [8] and Rupe [39] that stachyose is the main oligosaccharide in most legumes. Soaking of the red haricot bean in distilled water resulted in an insignificant decrease (*P* > 0.05) of 1.37% in the stachyose concentration of the red haricot beans. The stachyose concentration decreased from 1264.45 mg/100 g to 1247.18 mg/100 g. This was similar to the findings of Rupe [39] who reported a 1.25% decrease in stachyose concentration from 2400 mg/100 g to 2370 mg/100 g after 16 h of soaking in tap water. It was also in agreement with the findings of Niba and Rose [40] who reported that there were minimal changes in oligosaccharide content as a result of soaking of adzuki and mung beans. This explains why consumers still develop flatulence even after soaking of beans before cooking. The minimal decrease is attributable to leaching out of stachyose into soak water, since it is water-soluble [33]. Stachyose concentration decreased significantly when the soaked whole beans were fermented (Table 3). In the IF batch, the stachyose concentration decreased by 29.97% after 24 h of fermentation from 1247.17 mg/100 g to 873.35 mg/100 g. Increased fermentation time resulted in further decrease of 11.68% and 23.08% after 72 h and 120 h of fermentation. At the end of fermentation (120 h), the stachyose concentration in the IF batch was 593.33 mg/100 g.

In the SF batch, stachyose concentration decreased by 26.91% from 1247.17 mg/100 g to 911.61 mg/100 g after 24 h of fermentation. Further decrease of 39.95% and 14.59% in stachyose concentration occurred after 72 h and 120 h of fermentation (Table 3). At the end of 120 h of fermentation, the stachyose concentration was at 467.49 mg/100 g in the spontaneous fermentation batch. This is in agreement with the findings of Adewumi and Odunfa [7] and Granito et al. [8] who reported a decrease in stachyose concentration as a result of fermentation of *vigna ungiulata* beans and common beans (*Phaseolus vulgaris*), respectively. The highest decrease in stachyose concentration of the beans occurred between 24 h and 72 h of fermentation. However, even after 120 h of fermentation, an appreciable amount of stachyose reduction continued to occur. The spontaneously fermented beans recorded a slightly higher reduction of stachyose content compared to the inoculated batches. This is in agreement with the findings of Granito et al. [8] who reported that spontaneous fermentation removed more stachyose compared to controlled fermentation in Victoria beans.

## 4. Conclusion

Although soaking of red haricot bean lowers tannins, phytate, and raffinose content significantly, it has no significant effect on the stachyose. Yet this is the most abundant oligosaccharide in the bean. Fermentation of soaked whole red haricot beans results in increased significant reduction of tannin, phytate, raffinose, and stachyose concentrations. Fermentation of soaked red haricot bean lowers antinutrient and oligosaccharide content significantly. However, spontaneous fermentation of soaked whole red haricot bean results in significantly higher antinutrient losses. The highest antinutrient and oligosaccharide losses occur between 24 and 72 h of fermentation.

## Figures and Tables

**Table 1:** Effect of soaked whole red haricot beans fermentation on microbial growth (CFU/ML).

Fermentation time	*Lb. plantarum* BFE 5092 fermentation				Spontaneous fermentation			
	LAB	TVC	Coliform	Fungi	LAB	TVC	Coliform	Fungi
0 h	6.55 + 0.07^a^	6.84 ± 0.06^a^	5.58 ± 0.02^b^	ND	4.01 ± 0.01^a^	4.84 ± 0.01^a^	4.62 ± 0.14^b^	ND
24 h	8.57 ± 0.04^b^	8.64 ± 0.06^b^	8.08 ± 0.17^c^	ND	7.85 ± 0.01^b^	8.02 ± 0.08^b^	8.13 ± 0.38^d^	ND
72 h	8.34 ± 0.10^b^	8.64 ± 0.05^b^	5.54 ± 0.01^b^	ND	8.71 ± 0.06^c^	8.82 ± 0.03^c^	6.51 ± 0.20^c^	ND
120 h	8.31 ± 0.11^b^	8.39 ± 0.01^c^	3.19 ± 0.02^a^	ND	9.41 ± 0.26^c^	9.42 ± 0.02^d^	3.62 ± 0.30^a^	ND
*P* value	<0.0001	<0.0001	<0.0001	—	<0.0001	<0.0001	<0.0001	—

Values are means of triplicate determinations ± SEM. Means in the same column followed by the same superscript are not significantly different *P* > 0.05. Mean comparison for the treatments was done using the LSD test (*n* = 3). ND: not detected.

**Table 2:** Effect of soaking and fermentation on antinutrient content of whole red haricot bean.

	*Lb. plantarum* BFE 5092 fermentation		Spontaneous fermentation	
Treatment	Tannins mg/100 g	Phytates mg/100 g	Tannins mg/100 g	Phytates mg/100 g
Raw bean	333.58 ± 6.26^a^	502.99 ± 3.18^a^	333.58 ± 6.26^a^	502.99 ± 3.18^a^
Soaked	306.82 ± 0.41^b^	387.25 ± 10.31^b^	306.82 ± 0.41^b^	387.25 ± 10.31^b^
24 h fermentation	283.41 ± 3.13^c^	361.69 ± 2.54^c^	201.59 ± 2.18^c^	279.34 ± 3.17^c^
72 h fermentation	253.42 ± 3.62^d^	334.17 ± 4.55^d^	98.89 ± 6.19^d^	190.70 ± 3.78^d^
120 h fermentation	109.50 ± 6.38^e^	242.52 ± 3.32^e^	54.034 ± 4.00^e^	186.29 ± 2.98^d^
*P* value	<0.0001	<0.0001	<0.0001	<0.0001

Values are means of triplicate determinations ± SD. Means in the same column followed by the same superscript are not significantly different *P* > 0.05. Mean comparison for the treatments was done using the LSD test.

**Table 3:** Effect of soaking and fermentation on oligosaccharide content of whole red haricot bean.

	*Lb. plantarum* BFE 5092 fermentation				Spontaneous fermentation			
Treatment	Raffinose mg/100 g	% loss	Stachyose mg/100 g	% loss	Raffinose mg/100 g	% loss	Stachyose mg/100 g	% loss
Raw bean	72.27 ± 4.93^a^	0.00	1264.45 ± 58.90^a^	0.00	72.27 ± 4.93^a^	0.00	1264.45 ± 58.90^a^	0.00
Soaked	55.91 ± 1.98^b^	22.64	1247.18 ± 59.28^a^	1.37	55.91 ± 1.98^b^	22.64	1247.18 ± 59.28^a^	1.37
24 h fermentation	50.58 ± 5.14^bc^	9.52	873.35 ± 55.73^b^	29.97	50.22 ± 3.11^bc^	10.16	911.61 ± 29.24^b^	26.91
72 h fermentation	44.91 ± 6.74^c^	11.21	771.36 ± 43.38^b^	11.68	43.32 ± 3.74^c^	13.75	547.38 ± 11.77^b^	39.95
120 h fermentation	32.85 ± 5.05^d^	26.85	593.33 ± 7.35^c^	23.08	32.58 ± 3.95^d^	24.78	467.49 ± 13.36^c^	14.59
*P* value	<0.0001	—	<0.0001	—	<0.0001	—	<0.0001	—

Values are means of triplicate determinations ± SD. Means in the same column followed by the same superscript are not significantly different *P* > 0.05. Mean comparison for the treatments was done using the LSD test.

## Data Availability

The analyzed data used to support the findings of this study are included within the article.
